# Qualitative Analysis of Primary Fingerprint Pattern in Different Blood Group and Gender in Nepalese

**DOI:** 10.1155/2018/2848974

**Published:** 2018-01-18

**Authors:** Sudikshya KC, Niroj Maharjan, Nischita Adhikari, Pragya Shrestha

**Affiliations:** ^1^Department of Anatomy, National Medical College, Birgunj, Nepal; ^2^Department of Physiology, KIST Medical College, Lalitpur, Nepal; ^3^Department of Anatomy, Nepalgunj Medical College, Nepalgunj, Nepal; ^4^Department of Anatomy, Kathmandu University, Dhulikhel, Nepal

## Abstract

Dermatoglyphics, the study of epidermal ridges on palm, sole, and digits, is considered as most effective and reliable evidence of identification. The fingerprints were studied in 300 Nepalese of known blood groups of different ages and classified into primary patterns and then analyzed statistically. In both sexes, incidence of loops was highest in ABO blood group and Rh +ve blood types, followed by whorls and arches, while the incidence of whorls was highest followed by loops and arches in Rh −ve blood types. Loops were higher in all blood groups except “A –ve” and “B –ve” where whorls were predominant. The fingerprint pattern in Rh blood types of blood group “A” was statistically significant while in others it was insignificant. In middle and little finger, loops were higher whereas in ring finger whorls were higher in all blood groups. Whorls were higher in thumb and index finger except in blood group “O” where loops were predominant. This study concludes that distribution of primary pattern of fingerprint is not related to gender and blood group but is related to individual digits.

## 1. Introduction

Through decades of scientific research, the hand has come to be recognized as a powerful tool in the diagnosis of psychological, medical, and genetic conditions. The term dermatoglyphics was coined by Harold Cummins in 1926, which is used for the studies of epidermal ridges on the nonhairy part of palm, fingers, toes, and soles. He found that the configurations of ridge pattern are determined partly by heredity and partly by accidental or environmental influence, which produce stress and tension in their growth during fetal life. It has been accepted and adopted internationally [1]. It is based on the principle that the individual peculiarities of the patterns formed by the arrangements and distribution of the papillary or epidermal ridges on the fingertips are absolutely constant and persistent throughout life, from infancy to old age, and that the patterns of two hands do not resemble each other. Even the fingerprints of twins are not similar [2].

The pattern of dermal papillae determines the early development of the epidermal ridges. Early, in the fetal period, proliferation of the corium (dermis) forms papillary projections into the epidermis forming papillary ridges. The pattern of the papillary ridges in the hands is completely established between 11th and 24th weeks of gestation [3]. These features once formed remain permanent throughout the life of an individual except, in their dimensions, to commensurate the growth of an individual postnatally [4]. Over the past 150 years, dermatoglyphics has been a useful tool in understanding basic questions in biology, medicine, genetics, and evolution, in addition to being the best and most widely used method of personal identification.

Blood group system was discovered way back by Karl Landsteiner in 1901. Later “Rhesus” system was defined by Landsteiner and Wiener in 1937. A total of 30 human blood group systems are now recognized by the International Society of Blood Transfusion which vary in their frequency of distribution among various races of mankind. Clinically, only “ABO” and “Rhesus” groups are of major importance. “ABO” system is further classified as “A,” “B,” “AB,” and “O” blood groups according to presence of corresponding antigen in plasma. “Rhesus” system is classified into “Rh +ve” and “Rh −ve” according to the presence or absence of “D” antigen [5]. As the inheritance of dermatoglyphic patterns and ABO blood group is polygenic [6], the exact manner of inheritance of ABO blood group was revealed by Bernstein [7].

The available literatures have concluded that there is an association between distribution of fingerprint patterns, blood groups, and sexes. Suitable null and alternative hypothesis for the present study are as follows.

H_0_: there is no difference between fingerprints and “ABO” blood groups and “Rh” blood types in Nepalese males and females.
H_1_: there is significant difference between fingerprints and “ABO” blood groups and “Rh” blood types in Nepalese males and females.

## 2. Materials and Methodology

After obtaining the clearance from ethical and subject (research) committees, 300 Nepalese (Male = 150, Female = 150) were involved for data collection. They were informed about the procedures and consent was obtained.

### 2.1. Exclusion Criteria

- Individual with any hand deformity like permanent scars on fingers which may be congenital or acquired due to trauma on fingers
- Individuals suffering from any chronic skin disease, having worn fingerprints or extra or bandaged fingers

Details of individuals such as name, sex, and blood group were noted. In all of them (150 males and 150 females) blood group and Rh blood type were confirmed by slide agglutination method using antiserum “A,” antiserum “B,” and antiserum “D.” Each individual was asked to wash his/her hands thoroughly with soap and water to remove dirt and oil and dry them using a towel. He/she was then asked to press his/her fingertips on the stamp pad and then on respective blocks of Performa to transfer the fingerprint impression. Care was taken to avoid sliding of fingers to prevent smudging of the print. The fingerprint patterns were studied with the help of a magnifying lens and were identified into basic three patterns as follows: loops, whorls, and arches according to Galton's classification [8]. The distribution of fingerprint patterns in both hands of individuals and its relationship with sex and different ABO blood groups and Rh blood types were evaluated and analyzed by using chi square test and Bonferroni correction.

## 3. Results and Discussion

Table 1 shows the incidence of blood group “O” was highest followed by “B,” “A,” and “AB” blood groups, respectively, in both sexes. Similar results were reported by Bharadwaja et al. [9] and Prateek and Pillai [10]. On the other hand, A. A. Mehta and A. A. Mehta [11] and Desai et al. [12] observed the dominance of blood group “B” followed by blood groups “O” and “A.” The frequency of blood group “AB” was least in their study as well. The incidence of blood group “B” was observed as the highest in Pakistani by Khalid and Qureshi [13] and in Iran by Ghasemi et al. [14]. In Turkey, the incidence of blood group “A” (46%) was observed to be higher than blood group “O” (32%), while Europeans depicted the higher incidence of blood group “O” (43%) followed by blood groups “A” (40%), “B” (12%), and “AB” (7%) [5].

In this study, there was significantly higher incidence of Rh +ve (93.67%) subjects in all the blood groups as compared to Rh −ve (6.33%). This is in correlation with all the other studies done in various countries, that is, India [9, 10, 12], Libya [15], Pakistan [13], Iran [14], Nigeria [16], and Iraq [17].

Table 2 indicates that loops rule the chart followed by whorls and arches in both the sexes, which is in accordance with all previous works [9–25]. Chi square test reveals that the relation between primary patterns of fingerprints and sex is statistically not significant as *p* > 0.05. The null hypothesis is accepted and a conclusion is made that general distribution of primary fingerprint pattern is not related to gender.

In comparison between the two sexes, the loops are higher in incidence in females (52.4%) than in males (51.13%), but whorls and arches in males (43% and 5.87%, resp.) are more than in females (42.53% and 5.07%, resp.). This result is in correlation with the study of Sangam et al. [18] in the region of Andhra Pradesh except for the arches which are more in females (5.5%) than in males (3.7%) of their study. Similar to our study, Narahari et al. [19] observed the highest frequency of whorls (47.07%) in males and loops (60%) in females among the Khond community of Andhra Pradesh; Narahari and Padmaja [20] presented fingerprint data on Bondos of Malkangiri district of Orissa (India). They observed that their males possessed excess of whorls (48%) while the females displayed a high frequency of loops (48.66%). Contrary to the present study, Mehdipour and Farhud [21] observed in Iranian Muslims that the most common fingerprint patterns in males were loops (57.7%) when compared to females (56%).

Table 3 illustrates, in all the blood groups, there was predominance of loops ranging from 50.5% in blood group “AB” to 53.86% in blood group “O” with a difference of 3.36%. Our study is in resemblance with the study of A. A. Mehta and A. A. Mehta [11], who reported highest percentage of loops in blood group “O” (61.80%) and lowest percentage in blood group “AB” (47.27%). Shashikala and Ashwini [22] in Karnataka observed the percentage of loops was highest in blood group “AB” (61.76%) and lowest in blood group “O” (48.08%). This is contrary to the present study. However Deopa et al. [23] in Uttarakhand population reviewed highest percentage of loops in “B” blood group (62.2%) and lowest in “A” blood group (47.9%).

In present study, it was observed that percentage of whorls was highest in blood group “A” (45.39%) and lowest in blood group “O” (41.14%) with a range difference of 4.25% which resembles the study of Deopa et al. [23], that is, 45.7% in blood group “A” and 33.2% in blood group “O.” On the other hand Shashikala and Ashwini [22] reported highest incidence of whorls in blood group “O” (46.66%) and lowest incidence in blood group “AB” (24.11%) while A. A. Mehta and A. A. Mehta [11] observed highest frequency of whorls in blood group “B” (43.25%) and lowest frequency in blood group “O” (29.02%) with a range difference of 16.23%.

The incidence of arches in our study was highest in blood group “AB” (7%) and lowest in blood group “A” (3.95%) with a range of difference of 3.05%. Shashikala and Ashwini [22] and A. A. Mehta and A. A. Mehta [11] also observed highest percentage of arches in blood group “AB” but the lowest percentage of arches was reported in blood group “O” (5.25%) and blood group “B” (6.15%). However in both the studies the range differences of arches in various blood groups were higher than ours. Deopa et al. [23] reported the highest frequency of arches in “A” blood group (6.43%) and lowest frequency in “B” blood group (3.2%) with range difference of 3.23%.

In present study, blood group “A” reveals higher incidence of loops and whorls in females as compared to males. However, the arches are more common in males than in females. In blood group “B” incidence of loops is approximately same in both sexes. The frequency of whorls is more in males as compared to females. On the other hand, the frequency of arches is little more in females as compared to males. In blood group “AB” the frequency of loops in females is more than in males, while whorls and arches are more in males than in females. In blood group “O” the incidences of all types of fingerprints are approximately similar in males and females. Contrary to the present study, Manoranjitham et al. [24] observed higher incidence of loops and arches in females but whorls were more in males of blood groups “A” and “B” while in blood groups “AB” and “O” of their study loops and arches were more in females while whorls were more in males.

In all the above studies, including the present study, the general distribution of primary fingerprint patterns is of same order in individuals with ABO blood groups, that is, high frequency of loops followed by whorls and arches. Statistically, null hypothesis is accepted in all blood groups except “A” blood group. Hence, we can say primary fingerprint pattern is not related to ABO blood group in both males and females.

Table 4 shows percentage of loops was higher in Rh +ve blood group (52.14%) than Rh −ve blood group which correlates with the findings of Bharadwaja et al. [9] and A. A. Mehta and A. A. Mehta [11]. The percentage of whorls was more in Rh −ve blood group (48.94%) when compared to Rh +ve blood group (42.34%) which correlates with the study of Bharadwaja et al. [9] but contrary to the finding of A. A. Mehta and A. A. Mehta [11]. Percentage of arches in Rh +ve blood group (5.52%) is more than that of the persons belonging to Rh −ve blood group (4.74%). This did not correlate with Bharadwaja et al. [9] and A. A. Mehta and A. A. Mehta [11]. They reported higher incidence of arches in Rh −ve blood group.

Chi square test indicates the alternative hypothesis is rejected, so primary fingerprint pattern is also not related to Rh blood group.

Table 5 demonstrates that frequency of loops is higher in both the Rh +ve and Rh −ve individuals of ABO blood group, followed by whorls and arches, except in Rh −ve blood group “A” and Rh −ve blood group “B” where the incidence of whorls predominates the loops. In the present study both whorls and arches are not observed in Rh −ve blood group “AB,” while only arches are not reported in Rh −ve blood group “A.” Statistically it also shows the acceptance of null hypothesis except in “A” blood group.

In this study, in “A” blood group, the frequency of loops (52.39%) and arches (4.22%) was higher in Rh +ve blood group than in Rh −ve blood group (26% loops and 0% arches), but the incidence of whorls was more (74%) in Rh −ve blood group than in Rh +ve blood group (43.39%). This is statistically highly significant as *p* < 0.001. Bonferroni correction also suggests it is statistically significant as *p* < 0.0125. Our observations are in resemblance with the study of Fayrouz et al. [15] in Libyan population. In contrast to present study Bharadwaja et al. [9] and Umraniya et al. [25] in Gujrat population reported higher incidence of loops and arches in Rh −ve blood group and whorls in Rh +ve blood group, while Prateek and Pillai [10] and Desai et al. [12] in Karnataka population observed higher frequency of loops and whorls in Rh +ve blood group and arches in Rh −ve blood group.

In “B” blood group, percentile frequency of loops (51.05%) and arches (6.85%) was higher in Rh +ve blood group than Rh −ve blood group (loops 36.66% and arches 6.67%), while whorls (56.67%) were more in Rh −ve blood group than Rh +ve blood group (42.10%) in present study. This is supported by the study of Umraniya et al. [25] in Gujrat population. However Desai et al. [12] and Deopa et al. [23] in population of Uttarakhand observed higher percentage of loops and arches in Rh −ve blood group than Rh +ve blood group. Fayrouz et al. [15] reported higher frequency of loops and whorls in Rh −ve blood group while arches were more in Rh +ve blood group population. Our study is statically insignificant as *p* > 0.05; also Bonferroni correction also shows it is statistically not significant as *p* > 0.0125.

In “AB” blood group we observed only ten digits (one subject) of Rh −ve blood group having loops in all digits of both hands. Hence, the percentage frequency of loops is 100% which is obviously higher than Rh +ve blood group (47.89%). Interestingly whorls and arches were not found. So, there is total dominance of loop. This is in line with the work of Deopa et al. [23]. In contrast, Desai et al. [12] reported higher percentage of loops in Rh +ve blood group while more whorls and arches in Rh −ve blood group were reported. Fayrouz et al. [15] in Libyan population mentioned the higher percentage of all three patterns in Rh +ve blood group as compared to Rh −ve blood group. Their observations along our study by using chi square test and Bonferroni correction are statically insignificant as *p* > 0.05 and *p* > 0.025, respectively.

In present series of study in “O” blood group, there was higher percentage of loops (54%) and arches (7%) in Rh −ve blood group when compared to Rh +ve blood group (loops 53.86%, arches 4.79%). The whorls (41.35%) were common in Rh +ve blood group than Rh −ve blood group (39%). This is in resemblance with the results of Bharadwaja et al. [9] In contrast, Prateek and Pillai [10] and Desai et al. [12] reported higher incidence of loops and arches in Rh +ve blood group and whorls in Rh −ve blood group. Deopa et al. [23] observed higher incidence of loops in Rh −ve blood group and whorls and arches in Rh +ve blood group. Fayrouz et al. [15] found higher frequency of loops in Rh +ve blood group and whorls and arches in Rh −ve blood group. Their study was also statically insignificant as *p* > 0.05 as ours. Bonferroni correction also shows it is statistically not significant as *p* > 0.025.

On the basis of the fingerprint patterns of different digits, Table 6 shows the high frequency of loops in middle and little fingers of all the blood groups, that is, blood group “A” (m, 66.4%, and l, 67.7%), blood group “B” (m, 61.7%, and l, 69.4%), blood group “AB” (m, 62.5%, and l, 72.5%), and blood group “O” (m, 63.6%, and l, 72.1%). Bharadwaja et al. [9] and Shashikala and Ashwini [22] also reported high frequency of loops in middle and little fingers in all blood groups. Contrary to this, Fayrouz et al. [15] reviewed high frequency of loops in thumb, index finger, and ring finger in all blood groups in Libyan medical students. In the present study, frequency of whorls was higher in the ring finger of all blood groups, that is, blood group “A” (r, 64.4%), blood group “B” (r, 59.2%), blood group “AB” (r, 55%), and blood group “O” (r, 60.3%). This was in correlation with the observations of Bharadwaja et al. [9]. Fayrouz et al. [15] observed higher frequency of whorls in blood group “A” (r, 46.5%) and blood group “AB” (r, 46.9%) while loops were higher in ring fingers of blood group “B” (loops, 55.1%) and blood group “O” (loops, 45%). Shashikala and Ashwini [22] also reported high frequency of whorls in all the blood groups except in blood group “AB” where frequency of loops was highest (63.23%).

In our study, ring finger had high frequency of whorls in all blood groups; also thumb and index finger revealed higher frequency of whorls in blood groups “A,” “B,” and “AB,” that is, blood group “A” (t, 56%, i, 46%), blood group “B” (t, 54.5%, i, 44.3%), and blood group “AB” (t, 52.5%, i, 45%), but in blood group “O” loops were highest (t, 52.3%, i, 42.9%). Contrary to our study, Shashikala and Ashwini [22] reviewed high frequency of loops in index finger in blood groups “A,” “B,” and “AB” except blood group “O” where they reported high incidence of whorls (54.04%). Fayrouz et al. [15] observed higher frequency of loops in thumb and index finger of all blood groups in Libyan medical students. Bharadwaja et al. [9] observed predominance of whorls in index finger of blood groups “AB” (52%) and “O” (39.5%) and predominance of loops in blood group “B” (35.9%) but blood group “A” showed same frequency (41%) of both loops and whorls. In their study, in thumb, loops predominated in blood group “A,” 53%, blood group “B,” 60.4%, and blood group “O,” 57.8%, but in blood group “AB” whorls were highest (56%). This was in contrast to our study. However, Shashikala and Ashwini [22] observed high incidence of whorls in thumb of all the blood groups except blood group “AB.”

In the present study, frequency of arches was less than 10% in majority of cases but index finger of all blood groups depicted higher frequency of arches {blood group “A” (10.5%), blood group “B” (12.7%), blood group “AB” (25%), and blood group “O” (14.7%)}. Bharadwaja et al. [9] also observed higher incidence of arches in index finger of all blood groups as compared to other fingers including thumb. However, Fayrouz et al. [15] observed high frequency of arches in little finger of blood group “A” (14.4%), blood group “B” (23.1%), and blood group “O” (18.8%) except in blood group “AB” (21.9%) in which arches were highest in ring finger as compared to other fingers including thumb.

Both chi square test and Bonferroni correction show the rejection of null hypothesis. Hence we can conclude that primary fingerprint pattern distribution is related in individual digit in all blood groups.

## 4. Conclusion

The purpose of this study is to correlate the relationship between various patterns of fingerprints and “ABO” blood groups and “Rh” blood types in Nepalese males and females. Although we know that fingerprints are never alike and they never change from birth till death, this study is an attempt made to associate fingerprints with sex, different blood groups, and Rh blood types which may in turn enhance the authenticity of fingerprints in identification and forensic medicine and also can be used for possible prediction of certain diseases.

From the present study the following conclusions are drawn:

- Loops are the most commonly found fingerprint pattern and arches are the least common in both males and females and also in “ABO” blood groups.
- The frequency of loops is highest followed by whorls and arches in Rh +ve blood types, while the incidence of whorls is highest followed by loops and arches in Rh −ve blood types.
- Our results reveal highest incidence of loops in middle and little finger in all blood groups, while the whorls are commonly seen in ring finger in all blood groups. The frequencies of whorls are also highest in index finger and thumb in all blood groups except in blood group “O” where loops are frequently present.
- From this study we can conclude that distribution of primary pattern of fingerprint is not related to gender and ABO and Rh blood group, but its distribution is related to individual digits of both hands (Tables 2–6).

## Figures and Tables

**Table 1:** Distribution of males and females, ABO blood group, and Rh factor (*n* = 300).

Sex	A		B		AB		O		Total
	A +ve	A −ve	B +ve	B −ve	AB +ve	AB −ve	O +ve	O −ve	
Female	36	2	47	2	9	0	50	4	150
Male	35	3	48	1	10	1	46	6	150

Total	71	5	95	3	19	1	96	9	300

**Table 2:** General distribution of primary patterns of fingerprints in all digits of both hands of males and females (*n* = 3000).

Types of fingerprint patterns	Male	Female	Total
			%
Loops	767	786	1553
% within sex	51.13%	52.4%	51.76%

Whorls	645	638	1283
% within sex	43%	42.53%	42.77%

Arches	88	76	164
% within sex	5.87%	5.07%	5.47%

Total	1500	1500	3000
			100%

Statistics	*χ* ^2^ = 1.14, *p* = 0.5630, *p* > 0.05		

**Table 3:** Distribution of primary patterns of fingerprints in all digits of both hands among males, females, and ABO blood group (*n* = 3000).

Types of fingerprint patterns	Blood group A		Blood group B		Blood group AB		Blood group O	
	Male	Female	Male	Female	Male	Female	Male	Female
Loops	188	197	247	249	53	48	279	292
% within males and females in each blood group	49.47%	51.84%	50.41%	50.82%	48.18%	53.33%	53.65%	54.07%

Whorls	165	180	213	204	47	38	220	216
% within males and females in each blood group	43.42%	47.37%	43.47%	41.63%	42.73%	42.22%	42.31%	40.00%

Arches	27	3	30	37	10	4	21	32
% within males and females in each blood group	7.11%	0.79%	6.12%	7.55%	9.09%	4.45%	4.04%	5.93%

Total	380	380	490	490	110	90	520	540
%	100%	100%	100%	100%	100%	100%	100%	100%

Statistics	Chi^2^ = 20.062,		Chi^2^ = 0.944,		Chi^2^ = 1.39,		Chi^2^ = 2.24,	
	*p* = 0.000044,		*p* = 0.63,		*p* = 0.41,		*p* = 0.33,	
	*p* < 0.001		*p* > 0.05		*p* > 0.05		*p* > 0.05	

Bonferroni correction	*p* < 0.0125		*p* > 0.0125		*p* > 0.0125		*p* > 0.0125	

**Table 4:** Distribution of primary patterns of fingerprints in all digits of both hands among individuals of Rh positive and Rh negative blood group (*n* = 3000).

Types of fingerprint patterns	Rh +ve	Rh −ve
Loops (% within Rh)	1465 (52.14%)	88 (46.32%)
Whorls (% within Rh)	1190 (42.34%)	93 (48.94%)
Arches (% within Rh)	155 (5.52%)	9 (4.74%)

Total (%)	2810 (100%)	190 (100%)

Statistics	*χ* ^2^ = 3.17, *p* = 0.20, *p* > 0.05	

**Table 5:** Distribution of primary patterns of fingerprints in all digits of both hands among ABO blood group and Rh factor (*n* = 3000).

Types of fingerprint patterns	Blood group A		Blood group B		Blood group AB		Blood group O	
	Rh +ve	Rh −ve	Rh +ve	Rh −ve	Rh +ve	Rh −ve	Rh +ve	Rh −ve
Loops	372	13	485	11	91	10	517	54
% within Rh of blood group	52.39%	26%	51.05%	36.66%	47.89%	100%	53.86%	54%

Whorls	308	37	400	17	85	0	397	39
% within Rh of blood group	43.39%	74%	42.10%	56.67%	44.75%	0%	41.35%	39%

Arches	30	0	65	2	14	0	46	7
% within Rh of blood group	4.22%	0%	6.85%	6.67%	7.36%	0%	4.79%	7%

Total	710	50	950	30	190	10	960	100
%	100%	100%	100%	100%	100%	100%	100%	100%

Statistics	*χ* ^2^ = 18.18,		*χ* ^2^ = 2.64,		*χ* ^2^ = 10.32,		*χ* ^2^ = 1.01,	
	*p* = 0.0002,		*p* = 0.27,		*p* = 0.41,		*p* = 0.60,	
	*p* < 0.001		*p* > 0.05		*p* > 0.05		*p* > 0.05	

Bonferroni correction	*p* < 0.0125		*p* > 0.0125		*p* > 0.0125		*p* > 0.0125	

**Table 6:** Distribution of primary patterns of fingerprints in individual digits of both hands in subjects of different blood group (*n* = 300 × 2) (L = loops, W = whorls, and A = arches).

Individual finger	Blood Gr. A			Blood Gr. B			Blood Gr. AB			Blood Gr. O		
	(*n* = 760)			(*n* = 980)			(*n* = 200)			(*n* = 1060)		
	L	W	A	L	W	A	L	W	A	L	W	A
Thumb	63	85	4	83	107	6	18	21	1	111	98	3
% types of fingerprints within each blood group	41.4%	56%	2.6%	42.4%	54.5%	3.1%	45%	52.5%	2.5%	52.3%	46.2%	1.5%

Index	66	70	16	84	87	25	12	18	10	91	90	31
% types of fingerprints within each blood group	43.5%	46.0%	10.5%	43.0%	44.3%	12.7%	30%	45%	25%	42.9%	42.4%	14.7%

Middle	101	44	7	121	52	23	25	13	2	135	66	11
% types of fingerprints within each blood group	66.4%	29%	4.6%	61.7%	26.6%	11.7%	62.5%	32.5%	5%	63.6%	31.2%	5.2%

Ring	52	98	2	72	116	8	17	22	1	81	128	3
% types of fingerprints within each blood group	34.2%	64.4%	1.4%	36.7%	59.2%	4.1%	42.5%	55%	2.5%	38.3%	60.3%	1.4%

Little	103	48	1	136	55	5	29	11	0	153	54	5
% types of fingerprints within each blood group	67.8%	31.5%	0.7%	69.4%	28.1%	2.5%	72.5%	27.5%	0%	72.1%	25.4%	2.3%

Statistics	*χ* ^2^ = 84.19,			*χ* ^2^ = 100.39,			*χ* ^2^ = 38.44,			*χ* ^2^ = 122.87,		
	*p* < 0.00001,			*p* < 0.00001,			*p* < 0.00002,			*p* < 0.00001,		
	*p* < 0.001			*p* < 0.001			*p* < 0.001			*p* < 0.001		

Bonferroni correction	*p* < 0.0125			*p* < 0.0125			*p* < 0.0125			*p* < 0.0125		

## Additional Points

*Recommendations*. Similar study can be replicated on different ethnicity of people and different diseases. Studies can be done on a larger scale for quantitative study as well.
